# Supplementary material for: Generalizing the exact multipole expansion: density of multipole modes in complex photonic nanostructures
Source: Nanophotonics. 2022 Jul 14;11(16):3663–78. doi: 10.1515/nanoph-2022-0308 (PMC11501965; doi:10.1515/nanoph-2022-0308)
Supplement: Supplementary file 1 — Supplementary Material Details [file j_nanoph-2022-0308_suppl_001.pdf]

Supporting information for "Generalizing the exact multipole expansion: Density of multipole modes in complex photonic nanostructures"

Clément Majorel,[1] Adelin Patoux,[1, 2, 3] Ana Estrada-Real,[2, 4] Bernhard Urbaszek,[4] Christian Girard,[1] Arnaud Arbouet,[1] and Peter R. Wiecha[2, *]

[1] *CEMES-CNRS, Université de Toulouse, CNRS, UPS, 31000 Toulouse, France*
[2] *LAAS-CNRS, Université de Toulouse, 31000 Toulouse, France*
[3] *AIRBUS DEFENCE AND SPACE SAS, 31000 Toulouse, France*
[4] *INSA-CNRS-UPS, LPCNO, Université de Toulouse, 31000 Toulouse, France*

A. Exact multipoles vs. long-wavelength approximation

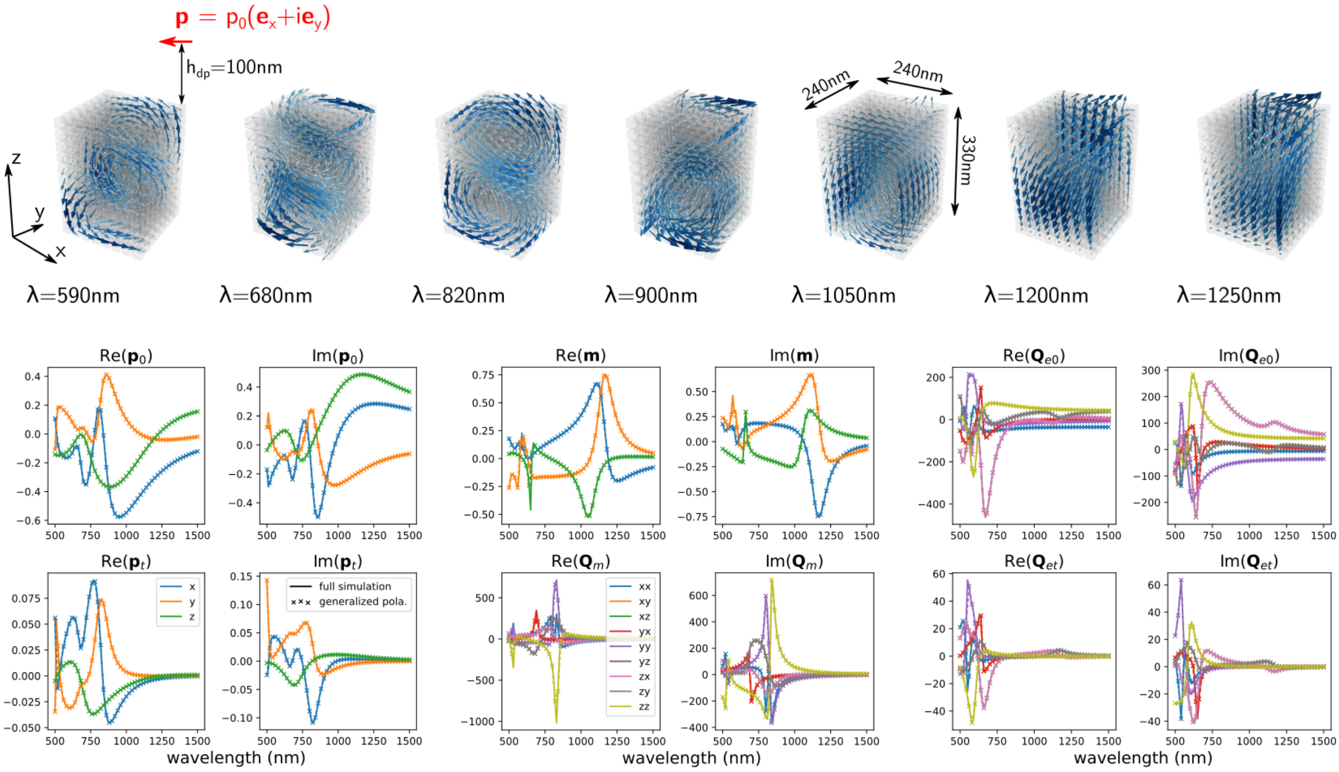

Figure S1. Demonstration of the accuracy of the generalized polarizabilities, compared to full field simulations. In order to obtain an inhomogeneous illumination, a chiral dipole emitter is located at a height of $h = 100\,$nm above the edge of a dielectric nano-cuboid of dimensions $240 \times 240 \times 330\,\text{nm}^3$ made of a lossless material ($n = 3.5$), placed in air ($n_{\text{env}} = 1$). The top figures show the real part of the internal field distribution at selected illumination wavelengths. The bottom plots show the real and imaginary parts of the effective multipole moments, obtained from the induced internal full fields following Ref. **?** (solid lines) as well as calculated using our generalized polarizability tensors (marker symbols). We plot the first order electric dipole (top left), toroidal dipole (bottom left), magnetic dipole (top center), magnetic quadrupole (bottom center), electric quadrupole (top right) and toroidal quadrupole (bottom right) modes. Plots show arbitrary units, normalized to the illumination dipole amplitude. Besides numerical noise the values obtained from both methods match exactly. The multipole amplitudes are in arbitrary units, relative to the illumination field amplitude.

* e-mail : pwiecha@laas.fr

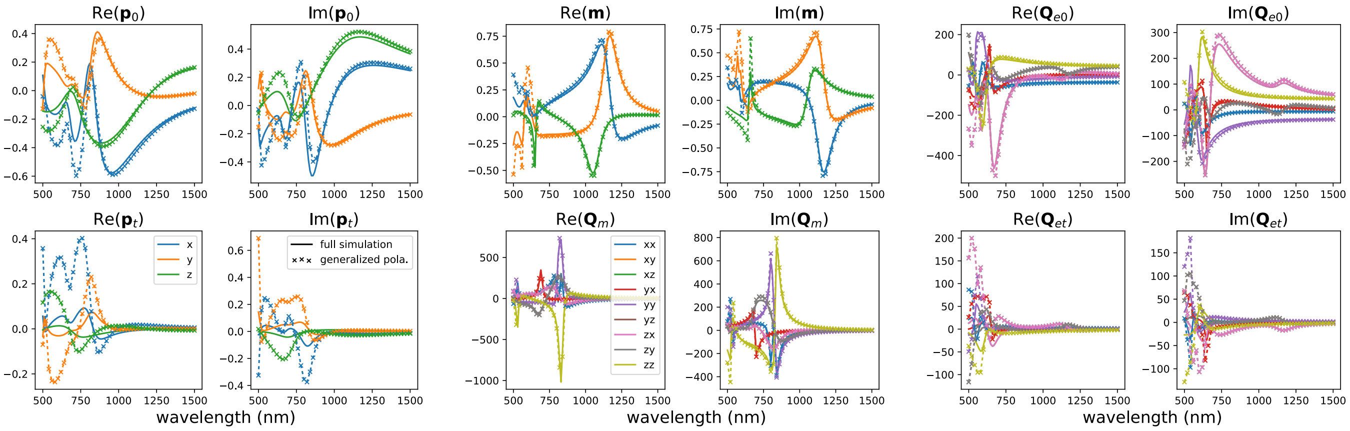

Figure S2. Same as figure S1, but with the long-wavelength approximation for calculation of the generalized polarizabilities. In particular at shorter wavlengths the error is drastically increasing, while the long wavelength approximation remains accurate.

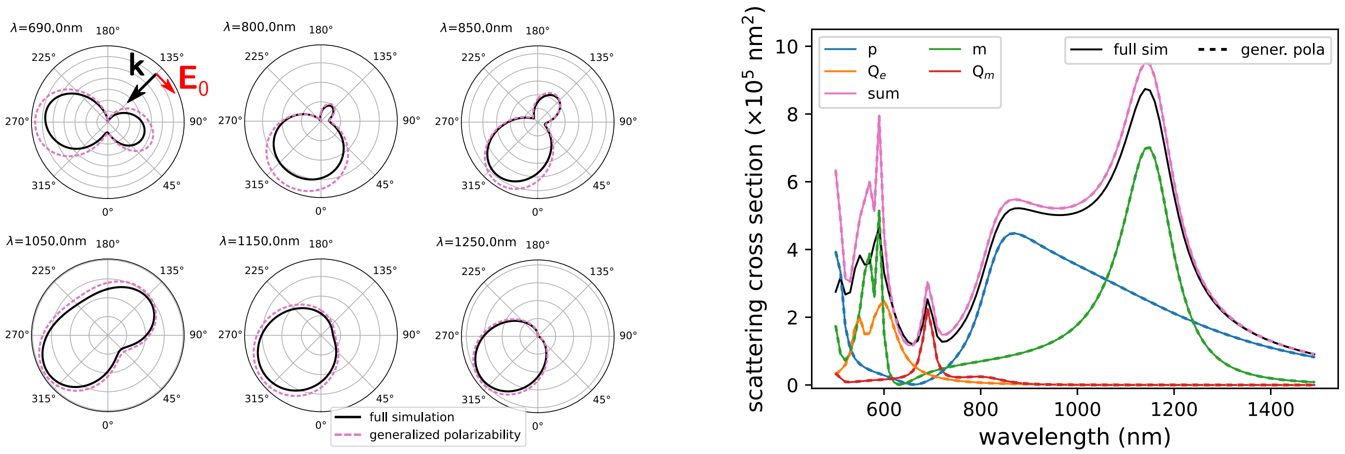

Figure S3. Same as figure 3 in the main text, but using the long-wavelength approximation for calculation of the multipole expansion and the generalized polarizabilities. In particular at shorter wavelengths the error is drastically increasing, while the long wavelength approximation remains relatively accurate at the red side of the spectrum. Still, an offset is deteriorating the accuracy already starting from the first resonance at around 1200nm.

B. Component-wise density of multipole modes

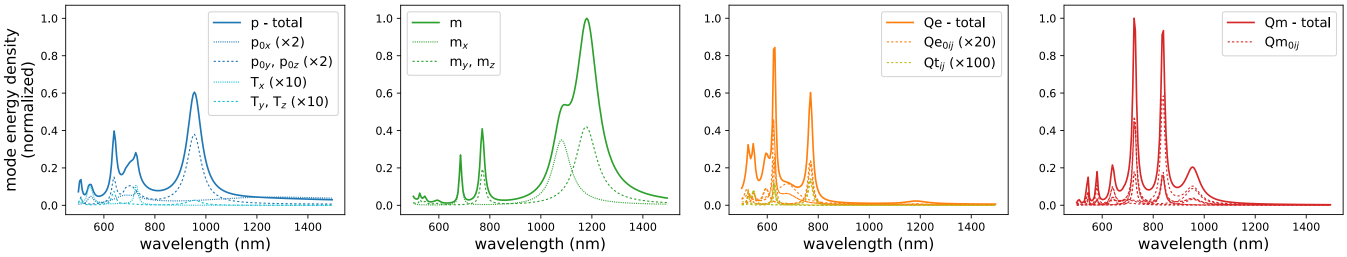

Figure S4. The same dielectric spheroid as discussed in the main text figures 4 and 5 is considered. Spectra are shown of the integrated Frobenius norms of the generalized polarizability sub-tensors, corresponding to individual components of the induced multipole moment. The spectra correspond hence to the mode density of the individual components of the multipole moments. In the quadrupole spectra the 9 tensor components are not labeled, the purpose of the figure is rather illustrative to show the capability of the method to resolve the different components.

C. Examples of excitation with "ideal" illumination field

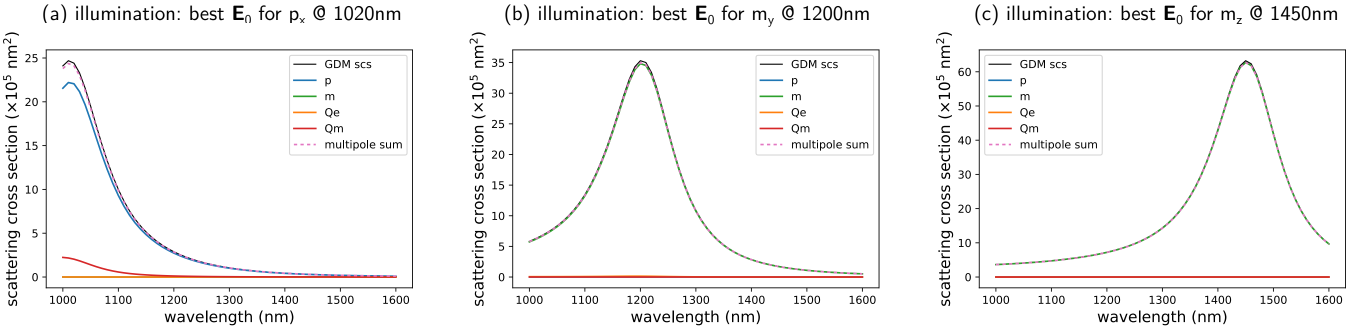

Figure S5. The same dielectric disc as shown in the main text figure 6 is considered. Spectra are calculated with an illumination corresponding to the ideal field distribution inside the structure for excitation of (a) an electric dipole moment along x at $\lambda_0 = 1020\,\text{nm}$, (b) a magnetic dipole moment along y at $\lambda_0 = 1200\,\text{nm}$, and (c) a magnetic dipole moment along z at $\lambda_0 = 1450\,\text{nm}$. The optimum illumination field described by the generalized polarizabilities induces almost purely the according mode. The scattering is normalized to the illumination field's peak amplitude, which is set to $E_0 = 1$, identical to the plane wave amplitude used for the spectra in the main text. So while it does not make much sense to define scattering cross-sections for the here used fields, the obtained values are still quantitatively comparable to the results obtained with plane wave illumination.

D. Test of the near-field accuracy of the generalized polarizabilities inside the circumscribing sphere

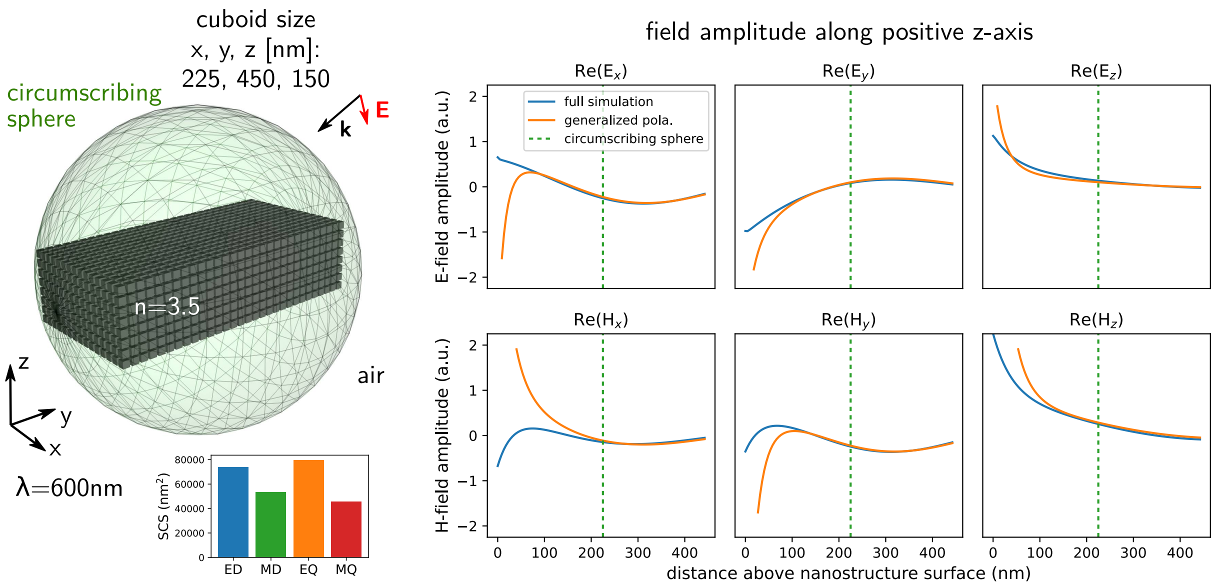

Figure S6. Test of accuracy of the generalized polarizabilities inside circumscribing sphere. The real part of electric (top row) and magnetic (bottom row) near-field amplitude is compared, calculated via full simulations (blue lines) and via the generalized polarizabilities (orange lines) using the dipole and quadrupole terms. The case of a very large structure with oblique incidence and diagonal polarization is chosen, in order to obtain an optical response with significant contributions of all four considered multipole terms (see histogram inset). Since the exact multipole decomposition is based on an expansion of the internal fields, the method remains accurate to a certain extent also inside the sphere containing the entire nanostructure. However, higher order terms contribute strongly to the very close near-field. Therefore, since only dipole and quadrupole terms are considered here, the accuracy is limited in the close vicinity of the structure.

E. Generalized polarizability vs. point polarizability

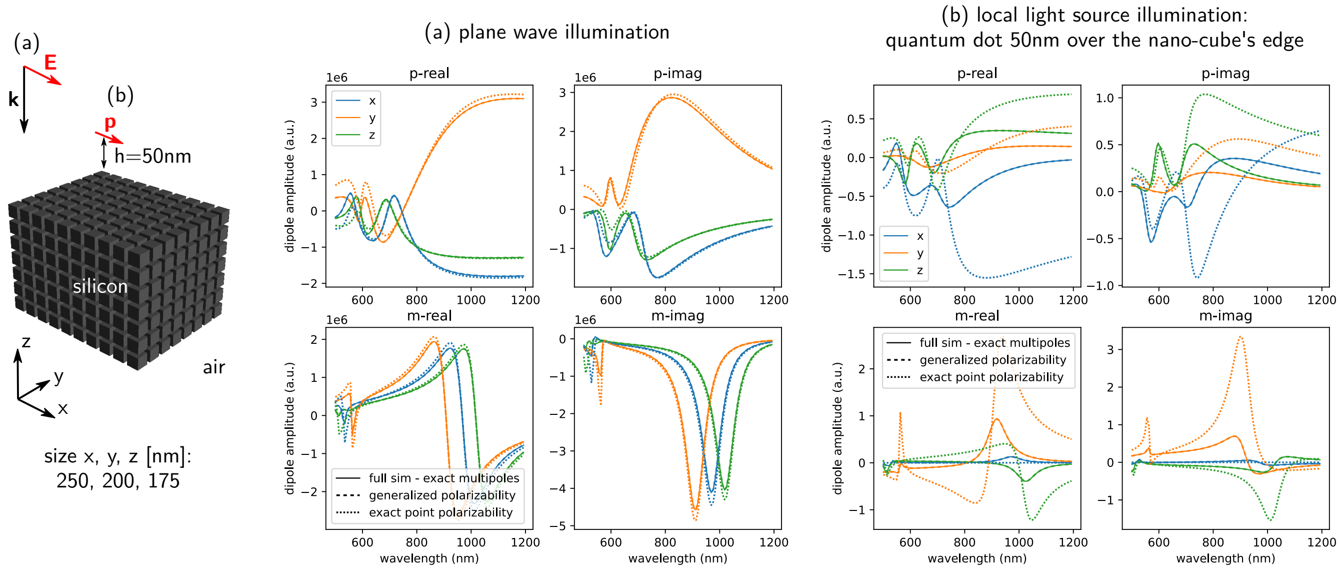

Figure S7. Comparison of the electric and magnetic dipole modes calculated via the exact multipole expansion from the full field simulations (solid lines), using the generalized polarizability formalism (dashed lines, this work) and a point-polarizability model based on exact multipoles, as introduced in [J. Mun et al., ACS Photonics 7(5), 1153-1162 (2020)] (dotted lines). The test structure is a silicon cuboid in air, illuminated (a) by an x polarized, normally incident plane wave and (b) by a local emitter oriented along x, placed 50 nm above the edge of the cuboid. The generalized polarizabilities reproduce identically the exact multipole decomposition. On the other hand, while the point-polarizability works well for a plane wave (on which the underlying assumptions rely), it totally fails at describing the response to the very inhomogeneous field distribution of a local light source.
